# Abnormal Dosage of Ultraconserved Elements Is Highly Disfavored in Healthy Cells but Not Cancer Cells

**DOI:** 10.1371/journal.pgen.1004646

**Published:** 2014-10-23

**Authors:** Ruth B. McCole, Chamith Y. Fonseka, Amnon Koren, C.-ting Wu

**Affiliations:** 1 Department of Genetics, Harvard Medical School, Boston, Massachusetts, United States of America; 2 Biological and Biomedical Sciences PhD program, Harvard Medical School, Boston, Massachusetts, United States of America; 3 Program in Medical and Population Genetics, Broad Institute of Harvard and MIT, Cambridge, Massachusetts, United States of America; University of California, San Francisco, United States of America

## Abstract

Ultraconserved elements (UCEs) are strongly depleted from segmental duplications and copy number variations (CNVs) in the human genome, suggesting that deletion or duplication of a UCE can be deleterious to the mammalian cell. Here we address the process by which CNVs become depleted of UCEs. We begin by showing that depletion for UCEs characterizes the most recent large-scale human CNV datasets and then find that even newly formed *de novo* CNVs, which have passed through meiosis at most once, are significantly depleted for UCEs. In striking contrast, CNVs arising specifically in cancer cells are, as a rule, not depleted for UCEs and can even become significantly enriched. This observation raises the possibility that CNVs that arise somatically and are relatively newly formed are less likely to have established a CNV profile that is depleted for UCEs. Alternatively, lack of depletion for UCEs from cancer CNVs may reflect the diseased state. In support of this latter explanation, somatic CNVs that are not associated with disease are depleted for UCEs. Finally, we show that it is possible to observe the CNVs of induced pluripotent stem (iPS) cells become depleted of UCEs over time, suggesting that depletion may be established through selection against UCE-disrupting CNVs without the requirement for meiotic divisions.

## Author Summary

Ultraconserved elements (UCEs) display a level of sequence conservation that has defied explanation. They are also dosage sensitive, being depleted from copy number variants (CNVs) in healthy cells. Here we address the process underlying this dosage sensitivity in order to gain insights into the way that UCE dosage affects cells. Our studies demonstrate that, in contrast to CNVs inherited by healthy individuals, cancer-specific CNVs are, as a rule, not depleted for UCEs and may even be enriched. Furthermore, by discovering that CNVs arising anew in the healthy, as opposed to diseased, body are depleted of UCEs, we obtain evidence that healthy cells may be responsive to changes in UCE dosage in a way that is disrupted in cancer cells. After examining CNVs over time in cell culture, we postulate that selection against UCE-disrupting CNVs in healthy cells acts rapidly, raising the surprising possibility of exploring in cell culture how UCE dosage sensitivity may explain ultraconservation. Our observations suggest that an understanding of the different responses of healthy and cancer cells to changes in UCE dosage could be harnessed to address genomic instabilities in cancer.

## Introduction

Ultraconservation came to light when Bejerano *et al*. reported that their comparison of the reference genomes of human, mouse, and rat had revealed an unexpected 481 orthologous genomic regions that are ≥200 bp in length and 100% identical in sequence [1], each of which is unique in the reference human genome [1], [2]. Ten years later, we still lack a compelling explanation for why these sequences, called ultraconserved elements (UCEs), have been so extremely conserved for hundreds of millions of years – neither enhancers, nor transcription factor binding sites, nor promoters, nor protein coding regions require such a high level of conservation [1], [3]–[8]. Despite this, and because roughly half of UCEs are intronic and one third are intergenic, a popular expectation is that UCEs will be found to embody important regulatory activities; indeed, they are thought to be maintained by purifying selection [9]–[16], and numerous UCEs are able to direct tissue-specific transcription when coupled with a reporter construct, while some have been shown to function endogenously as enhancers [4], [17]–[24]. UCE sequences can also contain various transcription factor binding motifs [6], [25] and bind multiple transcription factor proteins [26]. Ultraconservation could also be explained by a mechanism of comparison between pairs of UCEs. Here, the two copies of each UCE in a diploid cell, one on each of two homologous chromosomes, physically interact and then undergo sequence comparison, wherein discrepancies in DNA sequence or copy number, or disruptions of genome organization that compromise interactions, would be sensed and result in loss of fitness through disease or reduced fertility [2], [25], [27]. Such a mechanism would, over time, tend to cull away variants in UCE sequence or copy number, maintaining the extreme DNA conservation that characterizes UCEs. Importantly, there is growing evidence for the potential of homologous chromosomal regions to support at least transient, if not extensive, pairing in somatic cells [28]–[38] as well as in meiotic cells.

Interestingly, we and others have found that UCEs are much less likely to be deleted or duplicated via copy number variants (CNVs) in healthy individuals than would be expected by chance [2], [25], [39], consistent with their depletion from segmental duplications [2] and remarkable resistance to loss from mammalian genomes [40]. In contrast, they are enriched in 26 deletions and duplications representing 200 patients with neurodevelopmental disorders [41]. An association between UCEs and disease was also demonstrated [42] in a study that assembled a database of ‘cancer-associated genomic regions’ from a literature search for terms associated with cancer [43], and several publications have highlighted possible roles for the transcription of specific UCEs in cancer [44]–[51].

In sum, the basis of ultraconservation remains unclear. Indeed, it has been suggested that UCEs represent nothing more than an unexceptional tail end of a distribution of conservation [4]. Regardless, the apparent dosage sensitivity of UCEs remains intriguing, especially in light of the dosage sensitivity of many genomic functions whose importance has been well established [52]–[55]. Therefore, leaving aside the specific issue of ultraconservation, this report focuses on the dosage sensitivity of UCEs, with special emphasis on the time frame in which it is sensed. It takes advantage of 37 datasets of CNVs representing whole genome array-based or sequence-based analyses, and it begins with a demonstration that the most recently published datasets of CNVs representing healthy individuals are depleted for UCEs and that this depletion is robust to the mammalian species used to define UCEs. Importantly, we see that even *de novo* CNVs, which could have passed through the germline meiotic process at most once, are depleted of UCEs. This implies that CNVs need not be inherited through multiple generations to be depleted of UCEs. We then examine CNVs that have arisen in the soma, specifically in cancer cells, and discover that they are overall not depleted for UCEs. What is the basis by which CNVs in healthy people are depleted of UCEs, whereas cancer-specific CNVs display the opposite propensity? One possibility is that CNVs formed in the soma differ from those that are inherited across generations. Alternatively, CNVs specific to cancer may occur in positions that differ from those of CNVs found in healthy cells. To resolve this, we turn to CNVs that arise in healthy, as opposed to diseased, soma. We find that healthy somatic CNVs are depleted for UCEs, just as are CNVs inherited through the germline. This suggests that the profile of cancer specific CNVs reflects the diseased state, and not simply their somatic origin. Finally, to address how *de novo* and somatic CNVs of healthy individuals become depleted for UCEs, we examine the relationship, over time, between UCEs and CNVs in induced pluripotent stem (iPS) cells. Our results suggest that CNVs that have deleted or duplicated UCEs may be selectively removed from cell populations and that this process may underlie the UCE-depleted profile of CNVs present in healthy, but not cancer, cells.

## Results

### Depletion of UCEs from CNVs is seen in all inherited CNV datasets representing healthy individuals

**Figure 1:**
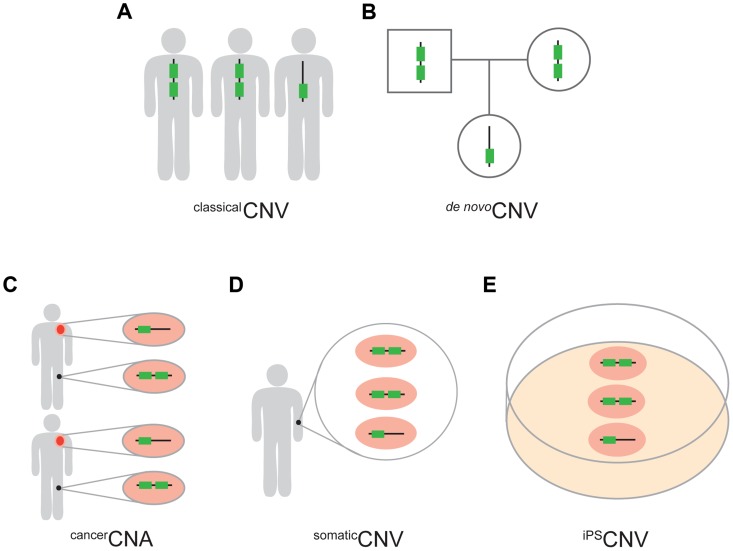
Five types of CNVs. (A) ^classical^CNVs are identified solely by variation among individuals in the copy number of genomic regions. (B) *^de nov^°*CNVs are present in an individual but not in the soma of either of the parents. (C) ^cancer^CNAs are copy number alterations that occur specifically in the cancer cells (orange) of an individual and, therefore, are absent from the healthy cells of the same individual (black). In this study we required ^cancer^CNAs to be recurrent between individuals. (D) ^somatic^CNVs are defined by regions that vary in copy number among the healthy somatic cells of an individual. (E) ^iPS^CNVs are defined by regions that vary in copy number within a population of iPS cells and which are not detectable in the fibroblast cells from which the iPS cells were derived.

We previously showed that UCEs are significantly depleted from CNVs in humans, with no overlap whatsoever between the positions of UCEs and CNVs in some cases, while, in other cases, the overlap was modest [2], [25]. There are three possible explanations that can account for these results: Firstly, CNVs could be completely excluded from forming in the vicinity of UCEs, and any overlap seen could be the result of inaccuracies in CNV mapping. Secondly, CNVs could be less likely to form in the vicinity of UCEs. Thirdly, CNVs may form in the vicinity of UCEs as much as expected by chance, but selective processes may then remove these CNVs from populations because they are deleterious, resulting in a depleted CNV profile over time. To help distinguish between these possibilities, we began our studies by determining whether, and to what extent, UCEs are depleted from six recent large scale high quality datasets of predominantly inherited CNVs representing healthy individuals (Matsuzaki *et al*. [56], Shaikh *et al*. [57], Conrad *et al*. [39], Drmanac *et al*. [58], Durbin *et al*. [59], and Campbell *et al*. [60]), including those obtained through next-generation sequencing [58], [59]. In order to facilitate comparison between the current and earlier studies, we also included two datasets that had been previously examined (Jakobsson *et al.* [61] and McCarroll *et al*. [62]). We call all these CNVs, which were discovered in healthy individuals without being specified as somatic or germline in origin, ^classical^CNVs (Fig. 1A). The eight individual ^classical^CNV datasets consist of between 1,183 and 46,716 regions and encompass between 0.83% and 45.25% of the human genome, a range in genome coverage that is not unexpected for datasets produced by studies that differ widely in their detection methods and sensitivity and in the number of subjects included. The datasets were considered individually as well as combined into a pooled ^classical^CNV dataset consisting of 43,727 CNV regions and covering 51.37% of the human genome (for more details, see Table S1).

Regarding the UCEs, the majority of our analyses were carried out with a set of UCEs we had previously defined [2]. This set of UCEs consists of sequences that are ≥200 bp in length and identical between the reference genomes of human, mouse, and rat (HMR), or of human, dog, and mouse (HDM), or of human and chicken (HC), producing a set of 896 (HMR-HDM-HC UCEs) UCEs in total [2]. We also generated two new UCE datasets without involvement of the human genome in order to ascertain whether the depletion of UCEs from CNVs is robust to the inclusion of UCEs selected without involvement of, and thus without perfect sequence identity to, sequences in the human genome. This strategy defined 527 UCEs using the reference genomes for dog, mouse, and rat (DMR) and another 1,696 UCEs using the reference genomes for cow, dog, and horse (CoDHo), all while maintaining the length and identity requirements of ≥200 bp and 100%, respectively (Figure S1, Methods). As the DMR and CoDH datasets involve only three species, while our original HMR-HDM-HC dataset involved four mammalian and one bird species, we assembled one additional dataset of 481 UCEs, this one using just the three reference genomes of human, mouse, and rat (HMR), as did Bejerano *et al*. when they defined the first UCE dataset [1]. Each of these four UCE datasets was studied in its entirety and, to parallel earlier work, subdivided into intergenic, intronic, and exonic subclasses; such earlier studies demonstrated that depletion is driven primarily by the intronic and intergenic UCEs, with evidence for that depletion being due to UCEs, *per se,* rather than flanking genetic regions or genes [2], [25].

Using a protocol established in earlier studies, we then determined whether UCEs are depleted in CNV datasets [2], [25]. We compared the observed amount of overlap in base pairs between a set of CNVs and a set of UCEs to the expected overlap, as determined by a randomly placed set of elements matched to UCEs in terms of element number and length. In particular, the elements of the matched set were placed randomly on the genome 1,000 times, and the overlap between the random elements and CNVs was calculated each time, thus producing a distribution of the randomly generated expected overlaps. To provide a measurement of the difference between the distribution of expected overlaps and the observed overlap, we reported the proportion of expected overlaps that were equal to, or more extreme than, the observed overlap. The distribution of expected overlaps was assessed for normality using the Kolomogorov-Smirnov (KS) test, and the associated KS P-value is included in all supplementary tables. Normality was observed in 263 of 318 (83%) of analyses and, whenever observed, the distribution of expected overlaps was compared to the observed overlap using a Z-test, wherein a significant result, together with a ratio of observed overlap to mean expected overlap (obs/exp) falling below 1.0 indicated significant depletion. Such an outcome would mean that the overlap between UCEs and CNVs is significantly lower than would be expected by chance, given the number, size, position, and genome coverage of the CNVs at hand. In cases where normality was not observed, we noted this in the text and reported only the obs/exp ratio and the proportion of expected overlaps that were equal to, or more extreme than, the observed overlap. This protocol ensured that each analysis was tailored to its own CNV dataset, enabling the meaningful comparisons of datasets that differ in terms of CNV number, size distribution, position, and genome coverage.

For pooled ^classical^CNVs, significant depletion was observed for all UCE datasets, with all values for obs/exp falling below 1.0 (P-values from <1.0×10^−17^ to 0.001, obs/exp from 0.771 to 0.867, Table 1 and Table S2). All individual ^classical^CNV datasets with normally distributed expected overlaps also showed significant depletion (8.8×10^−15^ ≤P≤0.020, 0.000≤ obs/exp ≤0.887, see Table 2 for HMR-HDM-HC UCEs, and Table S2 for all UCE sets); in three analyses, namely those addressing the DMR UCEs with respect to the McCarroll 2008 ^classical^CNV dataset, the Durbin 2010 ^clasical^CNV dataset, and the Campbell 2011 ^classical^CNV dataset, depletion could not be ascertained because the expected overlaps were not normally distributed (Table S2). As in previous studies, some analyses yielded 0 bp of overlap between UCEs and CNVs (e.g., HMR-HDM-HC UCEs and McCarroll 2008 [62], Drmanac 2010 [58], and Campbell 2011 [60]), while others showed some degree of overlap, with obs/exp ratios ranging from 0.021 to 0.887. The presence of multiple high quality datasets with non-zero overlaps between UCEs and CNVs led us to reject the first explanation, wherein CNVs are completely excluded mechanistically from forming at UCE regions and any observed overlaps are due to errors in mapping CNVs.

**Table 1:** Depletion of UCEs from pooled ^classical^CNVs is robust to the species used to define UCEs.

	Observed overlap		Expected overlap (bp)		Result			
UCE type	Number of UCEs	bp	Mean	Standard deviation	Proportion	P-value	obs/exp	Outcome
HMR-HDM-HC	368	97394	126264	4149	0.000	1.7×10^−12^	0.771	Depleted
DMR	239	61992	71462	3045	0.000	0.001	0.867	Depleted
CoDHo	713	196689	245830	5773	0.000	<1.0×10^−17^	0.800	Depleted
HMR	211	55832	66356	2966	0.000	1.9×10^−4^	0.841	Depleted

HMR-HDM-HC: 896 UCEs representing the union of Human-Mouse-Rat, Human-Dog-Mouse, and Human-Chicken UCEs [2]. DMR: 527 Dog-Mouse-Rat UCEs. CoDHo: 1,696 Cow-Dog-Horse UCEs. HMR: 481 Human-Mouse-Rat UCEs. Proportion: of 1,000 expected overlap iterations, the number of times the expected overlap generated was equal to, or more extreme than, the observed UCE overlap (bp), divided by the total number of iterations, which was always 1,000. P-value: significance of whether the observed overlap (bp) differs from the expected overlaps, as determined by a Z-test. obs/exp: observed overlap (bp) divided by mean of expected overlaps (bp). Outcome: determined with a one-tailed test (α = 0.05).

**Table 2:** Depletion of UCEs is observed in all ^classical^CNV datasets examined.

	Observed overlap		Expected overlap (bp)		Result			
Dataset	Number of UCEs	bp	Mean	Standard deviation	Proportion	P-value	obs/exp	Outcome
Pooled ^classical^CNVs	368	97394	126264	4149	0.000	1.7×10^−12^	0.771	Depleted
Jakobsson 2008	17	3922	12625	1795	0.000	6.2×10^−7^	0.311	Depleted
McCarroll 2008	0	0	2944	921	0.000	0.001	0.000	Depleted
Matsuzaki 2009	17	4078	9440	1668	0.000	0.001	0.432	Depleted
Shaikh 2009	7	2187	12981	1950	0.000	1.5×10^−8^	0.168	Depleted
Conrad 2010	1	202	9453	1642	0.000	8.3×10^−9^	0.021	Depleted
Drmanac 2010	0	0	4346	1142	0.000	7.1×10^−5^	0.000	Depleted
Durbin 2010	343	90605	110499	4258	0.000	1.5×10^−6^	0.820	Depleted
Campbell 2011	0	0	2160	790	0.000	0.003	0.000	Depleted

The 896 HMR-HDM-HC UCEs are depleted from all ^classical^CNV datasets. Proportion, P-value, obs/exp, and Outcome, as described for Table 1.

Note that depletion was also observed in many datasets when UCEs were separately analyzed as intergenic, intronic, and exonic elements (see Materials and methods for details on categorization of UCEs by genic location), with the intergenic and intronic classes driving depletion overall and the larger HMR-HDM-HC and CoDHo datasets showing stronger depletion (Table S2). While depletion was also observed with exonic UCEs, it was somewhat less consistent as that found with intronic and intergenic UCEs. The agreement of these results with our previous studies demonstrates that the depletion of UCEs from CNVs is a robust phenomenon and, hence, not dependent on 100% sequence identity between humans and other chosen species, extending our earlier observations [2], [25]. Accordingly, except where noted, all subsequent analyses in this study used the 896 UCEs of the HMR-HDM-HC dataset.

### Newly formed *de novo* CNVs are depleted for UCEs

Having eliminated the first explanation for depletion of UCEs from CNVs, we turned our attention to the two remaining possible explanations, which are not necessarily mutually exclusive; that CNVs are less likely to form in the vicinity of UCEs, and/or CNVs involving UCEs result in loss of fitness and are subsequently culled from the population. As some CNVs are recent enough to be polymorphic between individuals and even mosaic within individuals [63]–[68], the latter explanation would further suggest culling to be a relatively rapid process. We addressed these possibilities by seeking situations in which CNVs are not depleted for UCEs. If found, they would argue against CNVs being less likely as a rule to form near UCEs and, in addition, might permit us to estimate how rapidly CNVs are culled when they do involve UCEs. Accordingly, we turned to *de novo* CNVs, which are regions of copy number variation that are present in the soma of an individual but not in the soma of either parent. Leaving aside the possibilities of false positive regions (discussed in the Materials and methods), the oldest of such variants could have formed in the germline precursors of a parent and therefore passed no more than once through a germline. The youngest of such variants would include those that formed in the soma of an individual and are therefore less than one generation old, with no involvement of the germline (Materials and methods). We reasoned that these CNVs, which we call *^de nov^°*CNVs (Fig. 1B), may be so recent as to not yet have been culled of deletions and duplications that involve UCEs, if indeed UCE depletion results from a culling mechanism. In contrast, all ^classical^CNV datasets considered thus far in this report likely contain CNVs of varied ages, ranging from very newly formed CNVs arising within an individual's soma, to CNVs that have passed through the germline across many generations.

Four *^de nov^°*CNV datasets satisfied our criteria for further study (Xu *et al*. [69], Itsara *et al*. [70], Malhotra *et al*. [71], and Sanders *et al*. [72], detailed in Table S1); they represent studies using primary tissues as the source of DNA and requiring each *^de nov^°*CNV to have been validated by a second, independent method, such as Sanger sequencing (Materials and methods). While these studies examined patients with schizophrenia [69], [71] or autism [70], [72], they also included healthy individuals as controls, and it is the CNVs from healthy individuals that we used for our analysis. One study [70] included asthmatic individuals as healthy controls, and we did likewise. Because the four *^de nov^°*CNV datasets are small in terms of genomic coverage (0.05%–0.45%, Table S1), falling below our 20 Mb minimum requirement (see Table S3 section A for further discussion), we aggregated them into a pooled *^de nov^°*CNV dataset, including 25 CNVs covering 0.93% of the human genome (Table S1). Remarkably, this set of *^de nov^°*CNVs is significantly depleted of UCEs (P = 0.044, obs/exp  = 0.395, Table 3, Table S4 section A). Having discovered that even newly formed CNVs are depleted of UCEs, it remained possible that CNVs may be mechanistically biased against forming in the vicinity of UCEs. We therefore extended our search for CNV datasets that are *not* depleted for UCEs by turning to studies of CNVs associated with disease.

**Table 3:** UCEs are depleted from pooled *^de nov^°*CNVs, enriched in pooled ^cancer^CNAs, and depleted from pooled ^somatic^CNVs and high passage ^iPS^CNVs.

	Observed overlap		Expected overlap (bp)		Result			
Dataset	Number of UCEs	bp	Mean	Standard deviation	Proportion	P value	obs/exp	Outcome
				***^de nov^°*** **CNVs**				
**Pooled ** ***^de nov^°*** **CNVs** a	**4**	**967**	**2447**	**866**	**0.024**	**0.044**	**0.395**	**Depleted**
				**^cancer^CNAs**				
**Pooled ^cancer^CNAs** b	**642**	**171554**	**157016**	**3963**	**0.000**	**1.2×10^−4^**	**1.093**	**Enriched**
TCGARN 2008	60	15670	16549	2091	0.345	0.337	0.947	Neither
Walter 2009	121	32312	29544	2652	0.143	0.148	1.094	Neither
Beroukhim 2010	172	46060	47042	3270	0.383	0.382	0.979	Neither
Taylor 2010	84	23669	19685	2384	0.058	0.047	1.202	Neither
TCGARN 2011	259	67447	57988	3689	0.005	0.005	1.163	Enriched
Curtis 2012	51	13893	11872	1869	0.141	0.140	1.170	Neither
TCGARN 2012 breast	156	42421	26852	2677	0.000	3.0×10^−9^	1.580	Enriched
TCGARN 2012 colon	26	6813	10016	1672	0.021	0.028	0.680	Neither
TCGARN 2012 squamous	218	58477	51127	3424	0.020	0.016	1.144	Enriched
Robinson 2012	60	16125	11569	1844	0.010	0.007	1.394	Enriched
Walker 2012	893	240548	233338	1433	0.000	2.4×10^−7^	1.031	Enriched
Zhang 2012	37	10028	12325	1934	0.120	0.118	0.814	Neither
TCGARN 2013	83	21061	28531	2709	0.001	0.003	0.738	Depleted
				**^somatic^CNVs**				
**Pooled ^somatic^CNVs** c	**487**	**130694**	**142562**	**4019**	**0.004**	**0.002**	**0.917**	**Depleted**
Forsberg 2012	23	6576	4195	1061	0.018	0.012	1.568	Enriched
Jacobs 2012	265	69836	70178	3831	0.459	0.464	0.995	Neither
Laurie 2012	264	69935	67605	3559	0.259	0.256	1.034	Neither
McConnell 2013	221	60353	70021	3747	0.005	0.005	0.862	Depleted
				**^iPS^CNVs**				
**Pooled low passage ^iPS^CNVs** d	**15**	**3522**	**3235**	**1001**	**0.358**	**0.387**	**1.089**	**Neither**
**Pooled medium passage^ iPS^CNVs** d	**13**	**3633**	**6006**	**1283**	**0.020**	**0.032**	**0.605**	**Neither**
**Pooled high passage ^iPS^CNVs** d	**6**	**1279**	**3912**	**1033**	**0.003**	**0.005**	**0.327**	**Depleted**
Hussein 2011 low passage	13	2926	3086	943	0.466	0.433	0.948	Neither
Hussein 2011 medium passage	12	3428	5196	1240	0.069	0.077	0.660	Neither
Hussein 2011 high passage	1	211	1980	761	0.001	0.010	0.107	Depleted
Laurent 2011 high passage	5	1068	1962	768	0.108	NA	0.544	NA

Here, we show the relationship between 896 HMR-HDM-HC UCEs and *^de nov^°*CNVs, ^cancer^CNAs, ^somatic^CNVs, and ^iPS^CNVs, reporting the results for pooled datasets as well as all individual datasets that met our requirement for 20 Mb of coverage (Table S3). Individual CNV and CNA datasets are named according to the first author and the year of the study.

a The pooled *^de nov^°*CNV dataset included datasets from Xu 2008 [69], Itsara 2010 [70], Malhotra 2011 [71], and Sanders 2011 [72], which were too small to be considered on their own.

b The pooled ^cancer^CNA dataset included all the ^cancer^CNA datasets listed in this table, except for Walker 2012 [87], which was excluded to avoid bias from its extensive coverage of the genome, and also included the datasets Bullinger 2010 [78], Nik-Zainal 2012 [85], Holmfeldt 2013 [89], and Weischenfeldt 2013 [91], which were too small to be considered on their own.

c The pooled ^somatic^CNV dataset included the four ^somatic^CNV datasets listed in this table as well as Piotrowski 2008 [63] and O'Huallachain 2012 [67], which were too small to be considered on their own.

d The pooled ^iPS^CNV datasets were comprised of CNVs from low, medium, and high passage iPS cells from the two datasets Hussein 2011 [100] and Laurent 2011 [98]. Proportion, P-value, and obs/exp, as described for Table 1. Outcome: determined with a one-tailed test (α = 0.05) for the pooled *^de nov^°*CNV dataset because dataset was analyzed prior to our discovery that CNVs can be enriched for UCEs; all other assessments of depletion or enrichment carried out with a two-tailed test (P≤0.025 in each tail for an overall α of 0.05). NA (not applicable): expected overlaps not normally distributed, precluding a Z-test.

It is tempting to compare the obs/exp ratio of 0.395 (Table 3) for depletion of HMR-HDM-HC UCEs from pooled *^de nov^°*CNVs to the equivalent obs/exp ratio of 0.771 (Table 2) for depletion from pooled ^classical^CNVs and conclude that UCE depletion from *^de nov^°*CNVs is more extreme than from ^classical^CNVs. Note, however, that the obs/exp ratios for the individual ^classical^CNV datasets varied from 0.000 to 0.820 (Table 2). Given this wide range of values, the obs/exp ratio for pooled *^de nov^°*CNVs of 0.395 is not remarkably low.

### Copy number changes in cancer cells are enriched for UCEs

Our prediction that deletions and duplications of UCEs would reduce fitness [2], [25] argued that diseased tissues might yield datasets that are not depleted of UCEs. Consistent with this argument, UCEs have since been correlated with CNVs associated with diseases, including neurodevelopmental disorders [41] and cancer [42]. Here, we determined whether deletions and duplications found specifically in cancer cells are depleted of UCEs. Because such copy number changes are specific for the diseased, as versus healthy, tissues of an affected individual, they are believed to represent somatic events and, to highlight this difference from ^classical^CNVs, they are called copy number alterations, or CNAs [73]. In this report, we use ^cancer^CNAs to denote CNAs that were found specifically in cancerous tissues, and, as explained below, were also recurrent in multiple patients (Fig 1C).

For quality control, we required that ^cancer^CNA datasets represent studies wherein cancer genomes were defined relative to the genome of healthy tissues from the same patient. This strategy maximized the likelihood that our ^cancer^CNA datasets reflect alterations that arose within the affected individuals' lifetimes and specifically in cancerous tissues, thereby minimizing inclusion of ^classical^CNVs. Additionally, as ^cancer^CNAs that are recurrent in multiple patients are considered more likely to be causal “drivers” of disease, while non-recurrent ones are more likely to be merely “passengers” [73], we only included recurrent aberrations in our ^cancer^CNA datasets, identified as such using the GISITC [74] or RAE [75] algorithms, or our own analyses of recurrence (Materials and methods).

In total, we assembled seventeen datasets from The Cancer Genome Atlas Research Network (TCGARN) *et al*. [76], Walter *et al*. [77], Beroukhim *et al*. [73], Bullinger *et al*. [78], Taylor *et al*. [79], TCGARN *et al*. [80], Curtis *et al*. [81], TCGARN *et al*. [82], TCGARN *et al*. [83], TCGARN *et al*. [84], Nik-Zainal *et al*. [85], Robinson *et al*. [86], Walker *et al*. [87], Zhang *et al*. [88], Holmfeldt *et al*. [89], TCGARN *et al*. [90], and Weischenfeldt *et al*. [91] representing 52 different forms of cancer, each including between 2 and 148 ^cancer^CNA regions and covering 0.03% to 90.15% of the genome (Table S1). To avoid confounding our analysis with whole chromosome anueploidies, which are common in cancer genomes, we also followed convention [73] and excluded any ^cancer^CNA region that is larger than 50% of the chromosome arm on which it resides. The datasets were analyzed individually, except for Bullinger 2010 [78], Nik-Zainal 2012 [85], Holmfeldt 2013 [89], and Weischenfeldt 2013 [91], which are too small to be considered on their own (Table S3). We also pooled all datasets except one to produce our pooled ^cancer^CNA dataset; the Walker 2012 [87] dataset was excluded because it covers 90.15% of the genome and was therefore considered too large to be combined informatively with other datasets. Conveniently, two studies, Curtis *et al.* [81] and Walker *et al.* [87], also assembled datasets of ^classical^CNVs identified in nondiseased tissue of the patients used to identify ^cancer^CNAs. While the Curtis *et al.* [81] ^classical^CNV dataset was too small to be examined by our methods (Table S3), we found significant depletion of the Walker *et al.* [87] ^classical^CNV dataset, which represents 1,841 regions and covers 42.11% of the genome (Table S1; P = 0.008, obs/exp  = 0.903, Table S4 section B). This result gave us further confidence in the quality of the ^cancer^CNA datasets.

Turning to the ^cancer^CNA datasets themselves, we then observed a striking contrast to ^classical^CNVs and *^de nov^°*CNVs: of the 13 individual datasets large enough to be examined individually, all but two failed to show depletion for UCEs, as did the pooled ^cancer^CNA dataset (Table 3 and Table S4 section B; the TCGARN 2012 colon dataset [83] and the TCGARN 2013 dataset [90] showed depletion with P = 0.028, obs/exp  = 0.680 and P = 0.003, obs/exp  = 0.738 respectively). Indeed, as the values for obs/exp rose above 1.0 for several datasets, we converted to a two-tailed test (P≤0.025 in each tail for an overall α of 0.05) to detect potential enrichment (obs/exp>1.0) as well as depletion (obs/exp <1.0) for UCEs and discovered that our pooled dataset as well as five individual ^cancer^CNA datasets are significantly enriched for UCEs (3.0×10^−9^ ≤P≤0.016, 1.031≤ obs/exp ≤1.580, Table 3 and Table S4 section B). Furthermore, one of the datasets that had previously shown depletion was no longer significantly depleted (TCGARN 2012 colon [83]; P = 0.028, obs/exp  = 0.680, Table 3 and Table S4 section B) when using a two-tailed test.

Importantly, large genome coverage and CNA size are unlikely to explain enrichment or loss of depletion of UCEs in ^cancer^CNA datasets, and three findings support this statement. First, the broad range of genome coverage for ^cancer^CNA datasets showing enrichment or loss of depletion (from 90.15% for Walker 2012 ^cancer^CNAs to 3.86% for TCGARN 2012 colon ^cancer^CNAs) overlaps that for datasets that are depleted of UCEs (from 51.37% for pooled ^classical^CNVs to 0.83% for Campbell 2011 ^classical^CNVs), arguing that genome coverage alone cannot easily account for our observations of enrichment or depletion (Tables 2 and 3, S1, S2, and S4). Second, depletion is maintained when the boundaries of each CNV of the Jakobsson 2008 ^classical^CNV and Campbell 2011 ^classical^CNV datasets are extended on each side by 4.0 and 2.5 Mb, respectively (P = 0.007, obs/exp  = 0.968 and P = 0.003, obs/exp  = 0.952, respectively), such that the 85.86% and 74.73% genome coverages of these enlarged datasets approach or exceed the genome coverages of the two largest ^cancer^CNA datasets (90.15% for Walker 2012 ^cancer^CNAs and 63.81% for pooled ^cancer^CNAs; Table S1), once again indicating that high genome coverage is highly unlikely to produce false signals of enrichment or loss of depletion (Table S3 section B). We note, however, that as the genome coverage of the Walker 2012 ^cancer^CNA dataset is extremely high and exceeds the genome coverage of the enlarged ^classical^CNV datasets, we cannot rule out some contribution of genome coverage to the enrichment of this specific dataset. Third, these analyses also reveal that depletion is maintained even when the median length of enlarged CNVs (3.485 Mb and 8.379 Mb for Jakobsson 2008 ^classical^CNVs and Campbell 2011 ^classical^CNVs, respectively) exceeds the largest median CNA size for any enriched ^cancer^CNA dataset in question (3.183 Mb for TCGARN 2012 squamous ^cancer^CNAs), demonstrating that observations of UCE enrichment are unlikely to be explained simply by median CNA size (Tables S1 and S3 section B).

Taken together, our observations reveal a feature that distinguishes the ^classical^CNV and *^de nov^°*CNV datasets from those of ^cancer^CNAs. While the former two are characterized by a depletion of UCEs, not only do the ^cancer^CNA datasets generally fail to show depletion, several are enriched for UCEs. This dichotomy may be explained by differences in the mutational landscapes and/or selective forces between healthy and cancer cells, with healthy cells displaying a bias against CNVs in the vicinity of UCEs, and cancer cells being biased toward disruption of UCEs by CNVs. Whether nondepletion and/or enrichment will prove to be a universal signature of ^cancer^CNAs remains to be determined, the depletion of UCEs from one ^cancer^CNA dataset (TCGARN 2013 [90]) suggesting that the story will be more complex, perhaps reflecting tissue or cancer specificity. At the least, our findings argue that the depletion of UCEs that characterizes many CNV datasets is unlikely to reflect an intrinsic inability, across all cell types, of CNVs to form in the vicinity of UCEs.

### Intronic UCEs drive the enrichment of UCEs in ^cancer^CNAs

We have also analyzed the enrichment of UCEs in ^cancer^CNA datasets while treating intergenic, intronic, and exonic UCEs separately (Table S4 section B). Of these three UCE classes, only the intronic UCEs are enriched in pooled ^cancer^CNAs (P = 9.4×10^−5^, obs/exp  = 1.140), the intergenic and exonic UCEs showing neither depletion nor enrichment (P = 0.153, obs/exp  = 1.045 and P = 0.446, obs/exp  = 1.007, respectively; Table S4 section B). At the level of the five individual ^cancer^CNA datasets showing enrichment, we observed enrichment for both intronic and intergenic, but not exonic, UCEs. To better understand the basis for enrichment, we focused on the enrichment observed for the pooled dataset and entered the coordinates of all intronic UCEs overlapping pooled ^cancer^CNAs into the gene ontogeny tool GREAT [92] (Materials and methods). This analysis revealed no enrichment in cancer-specific GO terms, suggesting that the enrichment of intronic UCEs in ^cancer^CNAs may not be due to disruption of oncogenes or tumor suppressor genes, *per se*, but to an advantage for cancer cells of disrupting UCEs in particular. Additionally, the majority of intronic UCEs are overlapped by the pooled ^cancer^CNA dataset (78% of 418 intronic UCEs and 80% of 181 genes containing intronic UCEs), suggesting the effect is spread across many UCEs and not attributable to a small subset of UCEs or genes. To investigate this further, we examined the sixteen individual datasets that form our pooled ^cancer^CNA dataset, and scored each UCE for the number of times it is overlapped by a ^cancer^CNA dataset (Table S5). The highest hit rate was six, and this for an intronic UCE that is the one and only UCE in the gene neurotrimin (NTM), which has not been associated with cancer. Furthermore, of 327 intronic UCEs overlapping ^cancer^CNAs, 124 (38%) are overlapped by only one ^cancer^CNA dataset. As such, it appears that the enrichment of UCEs in ^cancer^CNAs relies on a large number of UCEs, with no particular UCEs being disrupted in a wide variety of cancers.

### The correlation between UCE and ^cancer^CNA positions is independent of the position of genes, microRNAs, transcribed UCEs, and enhancers, GC content, and replication timing

**Figure 2:**
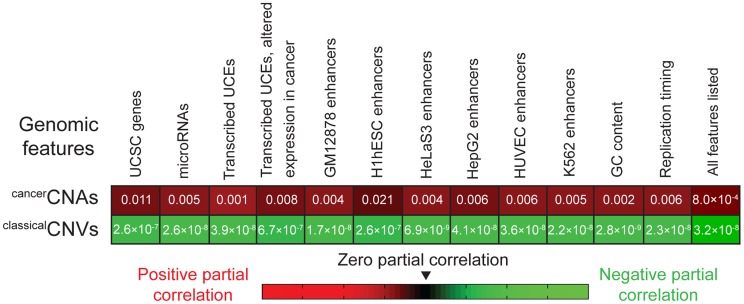
Partial correlation analyses. The positive correlation between the positions of UCEs and ^cancer^CNAs (first row) and the negative correlation between the positions of UCEs and ^classical^CNVs (second row) remain even after accounting for the correlation between the positions of UCEs and the genomic features listed across the top. P-values correspond to analyses in which the genome was divided into 50 kb windows and then assessed for the number of base pairs encompassed by the various genetic features within each window. Analyses using 10 kb and 100 kb bins also produced significant values across the board.

Finally, we applied partial correlation analyses (Materials and methods) to address whether the enrichment of UCEs in ^cancer^CNAs can be completely explained by the relative positioning of UCEs and another genomic feature, such as genes, or whether a positive relationship between the placement of UCEs and ^cancer^CNAs remains even when other genomic features are taken into account. We began by considering genes, dividing the genome into 50 kb windows and, within each window, scoring the number of base pairs encompassed by UCEs, ^cancer^CNAs, and genes. Next, we calculated the correlation between UCEs and ^cancer^CNAs, and then, using partial correlation analyses, statistically removed from this correlation any contribution that can be ascribed to the positions of genes. For comparison, we also ran parallel analyses examining the correlation between UCEs and ^classical^CNVs. As shown in the leftmost segment of Figure 2, the resulting partial correlation coefficient indicates that the correlation of UCEs with ^cancer^CNAs remains positive and significant, independent of the location of genes in the genome (P = 0.011). In contrast, and not surprisingly, we obtained a significant negative partial correlation between UCEs and ^classical^CNVs, indicating that the negative correlation of UCEs with ^classical^CNVs also cannot be explained by the position of genes (P = 2.6×10^−7^). Parallel analyses with window sizes of 10 kb and 100 kb gave similar results (0.004≤P≤0.014 for the enrichment of UCEs in ^cancer^CNAs and 2.2×10^−8^≤P≤1.9×10^−6^ for the depletion of UCEs from ^classical^CNVs).

Because microRNAs are associated with regions of the genome that are fragile in cancer as well as regions that are copy number variant in cancer cells [42], [43], [93], reviewed in [94], we asked whether the enrichment of UCEs within ^cancer^CNAs might simply be mirroring an effect that is centered on microRNAs. Using partial correlation analysis, we found that a significant positive correlation remains between the positions of UCEs and ^cancer^CNAs even when accounting for the position of microRNAs (P = 0.005). The positive correlation also remained when we controlled for the positions of transcribed UCEs and transcribed UCEs that show altered expression in cancer [42] (P = 0.001 and P = 0.008, respectively). As UCEs have been associated with enhancer function [4], [18]–[21], we examined whether a potential correlation between UCE and enhancer position could be driving the enrichment of UCEs in ^cancer^CNAs and/or their depletion from ^classical^CNVs. This analysis did not use enhancers that had been identified using sequence conservation [4] because a positive correlation between UCEs and such enhancers would be expected *a priori,* given that both the UCEs and enhancers would have been selected using similar criteria. Instead, enhancer regions were defined using the ‘enhancer’ annotations of ENCODE, which compiles chromatin and other modifications in six cell types [95]. We found that, even after accounting for the positions of enhancers, the positive correlation between UCEs and ^cancer^CNAs (0.004≤P≤0.021), as well as the negative correlation between UCEs and ^classical^CNVs (6.9×10^−9^≤P≤2.6×10^−7^), remained significant.

We also investigated the impact of GC content and differential replication timing across the genome, both of which have been found to be associated with the positions of ^classical^CNVs [96]. Here, again, the positive correlation of UCEs with ^cancer^CNAs remained significant in partial correlation analyses (P = 0.002 and P = 0.006, respectively), as did the negative correlation of UCEs with ^classical^CNVs (P = 2.8×10^−9^ and P = 2.3×10^−8^, respectively). Finally, we carried out partial correlation analysis while simultaneously controlling for *all* variables shown in Figure 2 and obtained a positive correlation between UCEs and ^cancer^CNAs (P = 8.0×10^−4^) as well as a negative correlation between UCEs and ^classical^CNVs (P = 3.2×10^−8^).

### Very newly formed, somatic CNVs are depleted for UCEs

Our data have thus far demonstrated significant depletion of UCEs from ^classical^CNVs and *^de nov^°*CNVs, while documenting a lack of depletion, or even a significant enrichment, in ^cancer^CNAs. One explanation for this difference might be that ^classical^CNV and *^de nov^°*CNV datasets represent generally healthy individuals while ^cancer^CNA datasets represent a diseased state. Alternatively, the difference could reflect an overall younger age of ^cancer^CNAs; whereas the ^cancer^CNAs we analyzed are most likely to have arisen somatically and not passed through a germline, some *^de nov^°*CNVs could have arisen in the germline of a parent, and many ^classical^CNVs are likely to have passed through many generations of germlines.

To further address the issue of CNV age, we examined CNVs that were established somatically but not in cancer cells, calling such variants ^somatic^CNVs (Fig. 1D). Here, we assembled ^somatic^CNV data from six publications: Piotrowski *et al*. [63], Forsberg *et al*. [64], Jacobs *et al*. [65], Laurie *et al*. [66], O'Huallachain *et al*. [67], and McConnell *et al.* [68]. In order to maximize the number of datasets of sufficient size for our analyses, we included CNVs obtained from the Jacobs *et al*. [65] and Laurie *et al*. [66] studies involving cancer patients, although we removed from consideration all CNVs representing individuals where the cancer-affected tissue was also tissue used to call ^somatic^CNVs (e.g. a person with leukemia whose blood was sampled to discover ^somatic^CNVs); the number of individuals falling into this excluded category amounted to only 16 (0.03%) from Jacobs *et al*. [65] and 7 (0.01%) from Laurie *et al*. [66]. We combined the six individual datasets into a pooled ^somatic^CNV dataset, consisting of 136 CNVs and covering 54.99% of the genome (Table S1). In contrast to ^cancer^CNAs, we find that the pooled ^somatic^CNV dataset is significantly depleted for UCEs (P = 0.002, obs/exp  = 0.917, Table 3 and Table S4 section C). These results show that the youthfulness of a CNV dataset does not necessarily predict an enrichment for UCEs. Furthermore, as they show that ^somatic^CNVs resemble ^classical^CNVs in terms of their depletion for UCEs, these observations suggest a potential similarity in the behavior of CNVs that pass through the germline and those that are formed in the soma. Note that three of the four individual datasets that were large enough to be analyzed on their own were not depleted of UCEs, with one being enriched: namely Forsberg 2012 [64], Jacobs 2012 [65], and Laurie 2012 [66]. In fact, these datasets, which consist of 5–104 CNVs and cover 2.04–27.10% of the genome (Table S1), do contribute to the depletion seen with the pooled ^somatic^CNV dataset. This becomes apparent when the three datasets are combined, leading the overall CNV coverage of the combined dataset compared to the three individual datasets to be increased by more than is the overlap of CNVs with UCEs (95% versus 93% for Forsberg 2012, 29% versus 22% for Jacobs 2012, and 32% versus 22% for Laurie 2012). Indeed, this combined dataset is itself depleted for UCEs (P = 0.011, obs/exp  = 0.902, Table S4 section C), explaining how these datasets, themselves not depleted for UCEs, contribute to the depletion seen in the pooled ^somatic^CNV dataset.

Turning to the ^somatic^CNV dataset that showed enrichment, Forsberg 2012 [64], we noted that all subjects in this dataset were over 60 years of age and therefore considered the possibility that advanced age may influence the relationship between UCEs and CNVs. We therefore examined the only two datasets of ^somatic^CNVs representing a wide range in sample ages, Jacobs 2012 [65] and Laurie 2012 [66] (Table S4 section C). Here we found an enrichment of UCEs in ^somatic^CNVs in individuals who are less than 60 years old (50 regions, 10.20% of the genome, P = 0.001, obs/exp  = 1.286) and neither enrichment nor depletion for those who are 60 or over (92 regions, 35.51% of the genome, P = 0.044, obs/exp  = 0.921). Hence, the enrichment of UCEs in the Forsberg 2012 [64] dataset is unlikely to be explained simply by the age of the subjects. Instead, our observations may reflect technical differences, such as sample selection and size, tissue-specificity of the mechanisms underlying depletion or enrichment of UCEs in CNVs, or the possibility of some ^somatic^CNVs representing tissues that are diseased, even if not diagnosed. Alternatively, a lack of depletion of UCEs from individual ^somatic^CNV datasets may reflect the fact that ^somatic^CNVs are very young and, perhaps also that they have not experienced passage through the germline, which may underlie and even be required for the more consistent depletion, and generally lower obs/exp ratios, observed with ^classical^CNVs (this study, [2], [25], [39]).

### iPS cells can establish UCE depletion from CNVs in culture

The depletion of UCEs from the pooled ^somatic^CNV dataset suggests that disrupting the dosage of UCEs may induce a fitness cost at the level of the individual somatic cell. Thus, we asked whether a signal consistent with selection of CNVs can be detected in cell culture. For example, although not proof of selection, lack of depletion at early time points giving way to significant depletion at later time points would be consistent with a selective loss of CNVs overlapping UCEs. To this end, we turned to iPS cell lines and examined their CNV profiles over time. To provide our analyses of different cell lines with a common starting point, we considered only those CNVs in iPS cells that were not detected in their matched parental cells, calling this subset ^iPS^CNVs (Fig. 1E). As we were interested in following the fate, rather than origin, of CNVs, we considered CNVs that arose *de novo* during cell growth in culture or as a result of the protocol for generating iPS cells [97]–[102] and those that were present in the parental cells at levels below the limit of detection [103]–[106] as equally relevant.

We required all studies to have genome-wide CNV profiles for iPS cell lines at multiple time points, or passage numbers, together with profiles for the matched parental cell line(s) from which the iPS cells were derived, and two studies satisfied our criteria: Hussein *et al*. [100] and Laurent *et al*. [98]. In the case of Hussein *et al.* [100], the dataset we assembled (Materials and methods) consisted of CNVs from 22 human iPS cell lines produced from 3 parental fibroblast lines, while for Laurent *et al*. [98] we assembled data for CNVs representing 36 iPS cell lines derived from 6 parental cell lines of various cell types. So that we could assay CNV profiles over time in cell populations, we pooled the ^iPS^CNVs from Hussein *et al*. [100] and Laurent *et al.* [98] into three categories, representing low, medium, and high passage, ensuring that the genome coverage of each category was sufficiently large for analysis. The low passage category represents cells from passages 4 and 5 (935 regions, 1.30% of the genome), the medium passage category covers passages 6 through 11 (1,071 regions, 2.39% of the genome), and the high passage category corresponds to passages 12 through 36 (300 regions, 1.63% of the genome) (Table S1). We also considered the Hussein *et al*. [100] and Laurent *et al.* [98] studies individually, seeking datasets corresponding to the passage numbers of the pooled datasets and yet still sufficiently large (Table S3) for our analyses; Hussein *et al*. [100] yielded low, medium, and high passage CNV datasets, and Laurent *et al*. [98] produced a high passage dataset (Table S1).

Intriguingly, we found that, while the pooled ^iPS^CNVs of low passage cells are not depleted for UCEs (P = 0.387, obs/exp  = 1.089), those of medium passage iPS cells trend towards depletion (P = 0.032, obs/exp  = 0.605), while those of late passage iPS cells give a clear signal of depletion (P = 0.005 obs/exp  = 0.327; Table 3 and Table S4 section D). As expected, given that the bulk of the pooled ^iPS^CNV data come from Hussein *et al*. [100] (Table S1), the results of our analysis of the Hussein *et al*. ^iPS^CNVs, alone, followed that of the pooled ^iPS^CNVs: Hussein 2011 low passage ^iPS^CNVs are not depleted for UCEs (P = 0.433, obs/exp  = 0.948), while Hussein 2011 medium passage ^iPS^CNVs trend towards depletion (P = 0.077, obs/exp  = 0.660), and Hussein 2011 late passage ^iPS^CNVs show significant depletion (P = 0.010, obs/exp  = 0.107; Table 3 and Table S4 section D). Although the Laurent 2011 high passage ^iPS^CNV analysis did not return expected overlaps that were normally distributed, precluding a P-value for depletion, this dataset nevertheless shows a low obs/exp ratio (obs/exp  = 0.544, Table 3 and Table S4 section D).

While the replication of our studies awaits the availability of additional ^iPS^CNV datasets of sufficient coverage and spanning considerable time frames, our findings thus far show that the CNV profiles of newly generated iPS cells can, at least under some circumstances, become depleted for UCEs over time. These observations are consistent with UCE-disrupting CNVs being under negative selection during iPS cell passage, with cells containing them being lost or out-competed over time. As such, they may explain why some CNVs may be selectively disfavored, even though they may not affect gene expression in the iPS cells [107]. How our observations interface with other studies documenting changes in CNV profiles over time in cell culture is difficult to assess, as these other studies represent a diversity of strategies for CNV analysis and differ among themselves in terms of the extent and direction of the changes in CNV abundance [97], [98], [100], [101], [103]. Furthermore, while our studies were focused on the overlap between CNVs and UCEs, these other studies were focused on the abundance, *per se*, of CNVs, which may not necessarily be correlated with depletion of UCEs. Nevertheless, our data indicate that depletion of UCEs from CNVs could occur without benefit of passage through the germline, suggesting that the mechanisms underlying depletion of UCEs from CNVs may be amenable to analysis in the laboratory.

## Discussion

In this study we provide evidence suggesting that a UCE-depleted CNV profile can be established in mitotically dividing cells without germline transmission. This finding, obtained with iPS cells, is consistent with our observation that, like ^classical^CNVs, *^de nov^°*CNVs and ^somatic^CNVs representing healthy individuals are depleted for UCEs as well. Drawing these findings together, we suggest that healthy human cell populations may be able to rapidly purge themselves of copy number variant regions involving UCEs. While this purging could involve the repair of CNVs, we find this unlikely, and instead favor the selective loss of cells containing CNVs that disrupt UCEs, such that the CNV profile of the remaining population of cells is depleted of UCEs.

In striking contrast to the situation in healthy cells, the CNVs of cancer cells are by and large not depleted of UCEs. This suggests an important and hitherto overlooked aspect of cancer genetics and invites the study of UCE depletion from CNVs into the realm of diseases that develop somatically, of which cancer is just one. Some diseased states may release cells from the dosage constraints of UCEs or even confer cellular advantages that outweigh the deleterious consequences of an imbalance of UCEs. Alternatively, release from the dosage constraints of UCEs may be a prerequisite or permissive step *en route* to disease. Our findings also highlight the possibility that some diseases associated with genomic instability involve instead, or in addition, a simple inability to cull away the normal burden of deleterious CNVs arising at a frequency that is not different from that found in healthy cells. In any case, lack of depletion of UCEs from a CNV dataset suggests that the cells contributing to the dataset may not represent the healthy state, having escaped the possible deleterious consequences of deleting or duplicating UCEs either because the mechanisms effecting such consequences were no longer in play or because the cells had acquired a means by which to circumvent them. With respect to ^cancer^CNAs, it may be that they arise when the mechanisms producing deleterious consequences are disabled or circumvented, their positions potentially influenced by the density of genes with either pro- or anti-proliferative functions [108], [109].

That ^cancer^CNV datasets can show an overall enrichment for UCEs is intriguing, especially since enrichment of UCEs in CNVs associated with disease has been observed in neurodevelopmental disorders [41]. In the case of cancer, it is unclear whether the enrichment we observe is on a continuum with loss of depletion or represents a subsequent or completely separate process. For example, release from the dosage constraints of UCEs may enable cancerous cells to benefit from growth advantages brought about by deletions or duplications of UCE-containing regions [45]–[50], [110]. This explanation is consistent with the observation that some transcribed UCEs can act as oncogenes [42], [47] or tumor suppressors [45] or, in the case of one UCE, intercellular signaling molecules within hepatocellular cancer [111]. An enrichment of UCEs in ^cancer^CNVs could also be explained if UCE dosage were directly or indirectly implicated in cell cycle control. Here, we presume that cellular detection of UCE dosage is coordinated with the cell cycle, since a cell doubles its ploidy as it traverses S-phase, and S-phase, itself, imposes a dosage imbalance that sweeps across the genome. As such, S-phase induced imbalances of UCEs could be used by a replicating cell to confirm that it is in S-phase and must continue to replicate its genome. If so, cells for which UCE dosage has been disrupted and, as suggested above, have also circumvented the deleterious consequences of aberrant UCE dosage, might be predisposed to continuously undergo replication and, hence, progress unrestrained through cell cycles. Of these, cells that are the most disrupted in UCE dosage, in other words enriched for the inclusion of UCEs in their CNVs, might be expected to show the strongest phenotype of unregulated growth and thus become cancerous.

The enrichment of UCEs in many ^cancer^CNA datasets may at first be difficult to reconcile with the depletion of UCEs from ^classical^CNVs, *^de nov^°*CNVs, ^somatic^CNVs, and ^iPS^CNVs; while cancer cells with abnormal UCE copy number appear unaffected or even advantaged, cells with abnormal UCE copy number may be disadvantaged in healthy individuals, this difference implying opposite impacts on proliferation, senescence, or apoptosis. Similarly, the mutational profiles of cancer cells may bias CNVs toward forming in the vicinity of UCEs, possibly conferring selective advantage, whereas the profiles of healthy cells may avoid such disruptions.

Whether the difference in UCE disruption by CNVs in cancer versus healthy cells is due to differences in mutational profiles, selective retention/loss of UCE-disrupting CNVs, or a combination of both, the dichotomy of CNV profiles with respect to UCEs between healthy and cancerous cells warrants further discussion. One explanation argues that even though cancer cells with disrupted UCE dosage may acquire a growth advantage, their presence is detrimental to the overall fitness of the individual. Hence, disruptions in UCE copy number such as those seen in cancer would not be predicted to endure in human populations, consistent with the UCE-depleted profile of ^classical^CNVs. The same argument cannot, however, be applied to *^de nov^°*CNVs, ^somatic^CNVs, or ^iPS^CNVs, because unlike ^classical^CNVs, these three categories of CNVs have not been subjected to selection at the level of the population. As such, the UCEs that are enriched in ^cancer^CNAs may differ from those that are depleted from *^de nov^°*CNVs, ^somatic^CNVs, or ^iPS^CNVs. This possibility can be further investigated when more *^de nov^°*CNVs, ^somatic^CNV, and ^iPS^CNV datasets become available.

Comparison of the locations, sizes, and sequences of UCEs, their potential differential inclusion in duplications or deletions, and other structural features may ultimately shed light on the basis for the enrichment of UCEs in some CNV datasets and the depletion of UCEs from others. As importantly, it may elucidate how loss or gain of a UCE could be sensed by the healthy cell and then translated into a deleterious consequence. At present, we favor a mechanism wherein the maternal and paternal copies of a UCE compare their sequences, possibly through pairing, because, by hypothesizing that any discrepancy between the homologs would trigger deleterious outcomes, this model offers an explanation for ultraconservation itself [2], [25], [27]. Such a pairing-based mechanism would contribute to genome integrity with respect to dosage and is compatible with the viability of mice that are homozygous for the loss of a UCE [112] (further discussion of heterozygous UCE deletions is presented in Chiang *et al.* [25]). Requirements for sensing and maintaining dosage in the genome are well studied (for examples, see [52]–[55]), and responses to dosage imbalances, flagged by improperly paired UCEs, could range from a growth disadvantage among cells to loss of individuals from a population through disease and, at the molecular level, from metabolic disruptions to deleterious mutational and epimutational changes. Intriguingly, mutation within and in the vicinity of UCEs that are no longer well paired with a homolog may predict that ultraconserved chromosomal regions might be enriched in *de novo* mutations. Such a prediction is aligned with an intriguing observation, wherein conserved sequences appear to occupy the more mutable parts of the human genome, at least with regards to *de novo* mutations ([113], [114], see also [115]). In particular, heterozygosity for a CNV that deletes or duplicates a UCE could enhance local rates of *de novo* mutation due to disruption of pairing and, if such mutations confer a selective disadvantage, they will be lost from the population, thus increasing mutation rates in the short term while promoting conservation of UCE sequence and dosage over longer time frames. It is also possible that, if the unpaired status of a UCE persists for an extended period of time, *de novo* mutations may not all be removed by selection and perhaps even accumulate. In such a situation, the DNA sequence of the UCE could decay, in which case the deleterious response to disrupted pairing (loss of fitness, e.g., disease and infertility) would vanish, explaining how UCEs can be lost, albeit rarely [40]. UCEs could also be disabled through epigenetic modification without disruption of UCE sequence. Here, too, the resultant lack of constraint on a UCE could lead to the decay of its sequence.

**Figure 3:**
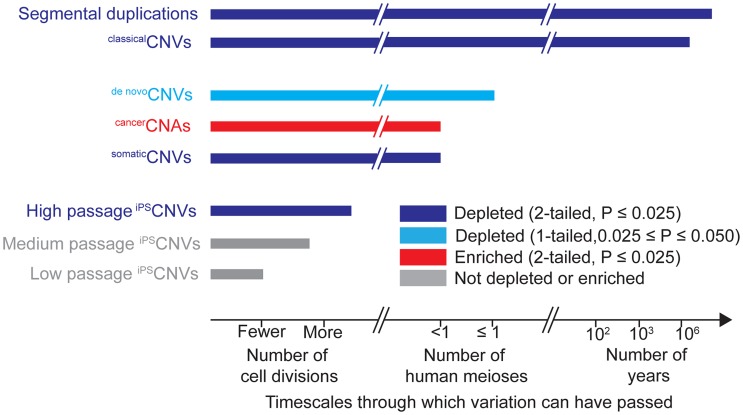
Timescales through which different types of genomic variation have been present and their relationships to UCEs.

Finally, our results also demonstrate that the depletion of UCEs from CNVs may be tractable to analysis in cell culture; whereas studies of UCEs have generally been conducted in the context of many human generations or evolutionary timescales, our findings demonstrate that depletion of UCEs from CNVs and possibly ultra-conservation, itself, are amenable to analyses spanning just a few cell generations (Fig. 3). Excitingly, understanding the relative contributions of CNV formation and selection pressure to UCE depletion in healthy cells and loss of that depletion in cancer cells should help reveal how cancer cells differ from healthy cells and, perhaps, how we may mitigate cancer phenotypes by inducing cancer cells to more closely resemble healthy cells. Indeed, if we understand the mechanisms by which UCE depletion is established in healthy cells, be it through selection against UCE-disrupting CNVs or otherwise, such mechanisms could be harnessed to purge a diseased tissue or individual of diseased cells, while leaving untouched cells whose CNV profiles do not disrupt UCEs. Such a strategy could prove even more powerful should UCEs embody a mechanism, perhaps through pairing, by which cells assess all types of genome rearrangements, distinguishing the deleterious from the benign or even beneficial.

## Materials and Methods

### UCE identification

Two new sets of ultraconserved elements were defined in this study: one between the reference genomes of cow, dog, and horse (builds: bosTau6, canFam2, and equCab2) and the other between the reference genomes of dog, mouse, and rat (builds: canFam2, mm9, and rn4). We also identified UCEs between human, mouse, and rat (builds: hg18, mm9, rn4), which are very similar to the UCEs identified in 2004 [1], although earlier builds were used to identify UCEs in that study. Pairwise alignments were found between each possible pair of genomes within the set of three, and elements with 100% basepair identity that were ≥200 bp in length were selected. We then mapped these regions to the hg18 human genome by BLAT (http://genome.ucsc.edu/cgi-bin/hgBlat), filtering out matches in the human genome that differed in length by more than 3 bp and were not unambiguously unique in the human genome. The hg18 orthologs of our new UCE sets were then used in our analyses. Coordinates for all UCEs are available in Table S2.

#### Classifying UCEs as intergenic, intronic, or exonic

UCEs were classified as intergenic, intronic, or exonic using the UCSC Known Genes track for hg18. If a UCE overlapped neither exons nor introns, it was designated intergenic. If a UCE did not overlap an exon but did overlap an intron by 1 bp or more, it was designated intronic. If a UCE overlapped an exon by 1 bp or more, it was designated exonic.

### Dataset acquisition and filtering

Table S1 provides detailed information for all CNV datasets, including the number of affected regions, median size of CNVs, genome coverage, discovery and validation platforms used, number of subjects, and coordinates. When necessary, coordinates were mapped to the hg18 genome build using the liftover utility provided by UCSC (http://genome.ucsc.edu/cgi-bin/hgLiftOver). In each CNV dataset, overlapping regions were collapsed to avoid counting the same region multiple times, leading to a final list of regions for each CNV dataset that may differ from the original set reported in the relevant publication. Additional information for the various CNV datasets can be found below.

#### ^classical^CNV datasets

Eight ^classical^CNV datasets were obtained from Jakobsson *et al.* [61], McCarroll *et al*. [62], Matsuzaki *et al*. [56], Shaikh *et al*. [57], Conrad *et al*. [39], Drmanac *et al*. [58], Durbin *et al*. [59], and Campbell *et al*. [60].

#### *^de nov^°*CNV datasets

Four *^de nov^°*CNV datasets were obtained from Xu *et al*. [69], Itsara *et al*. [70], Malhotra *et al*. [71], and Sanders *et al*. [72]. The identification of *^de nov^°*CNVs is exceptionally vulnerable to errors, because each *^de nov^°*CNV requires two negative results (the CNV is not detected in either parent). For example, if a CNV is missed in the parents, but is correctly detected in a child, it will be incorrectly designated a *^de nov^°*CNV. Additionally, the use of cell lines to detect *^de nov^°*CNVs may produce artifacts, as CNVs may arise *de novo* within a cell line [70], [116], [117]. For these reasons, we only studied a *^de nov^°*CNV if it had been identified using DNA obtained directly from primary tissue and independently verified.

#### ^cancer^CNA datasets

Seventeen ^cancer^CNA datasets were obtained from TCGARN *et al*. [76], Walter *et al*. [77], Beroukhim *et al*. [73], Bullinger *et al*. [78], Taylor *et al*. [79], TCGARN *et al*. [80], Curtis *et al*. [81], TCGARN *et al*. [82], TCGARN *et al*. [83], TCGARN *et al*. [84], Nik-Zainal *et al*. [85], Robinson *et al*. [86], Walker *et al*. [87], Zhang *et al*. [88], Holmfeldt *et al*. [89], TCGARN *et al*. [90], and Weischenfeldt *et al*. [91]. All data were filtered to remove any ^cancer^CNA longer than 50% of the length of the chromosome arm on which it resides. This was done to remove ^cancer^CNAs that result from losses of whole chromosomes or chromosome arms, events that we consider distinct from the smaller deletions and duplications considered in the present study.

We only considered recurrent ^cancer^CNAs, as they were more likely to be important for cancer causation or progression. In cases where published datasets had already been filtered for recurrent CNAs, we listed the algorithm used in Table S1. We did not further filter these datasets. The datasets of Bullinger *et al.* [78], Nik-Zainal *et al.* [85], Robinson *et al.* [86], Walker *et al.* [87], Holmfeldt *et al.* [89], and Weichenfeldt *et al.* [91] had not been pre-filtered for recurrent variants, and so, for these, we selected only ^cancer^CNA regions that were present more than once in the dataset. All these datasets except for that of Walker 2012 [87] were included in the pooled ^cancer^CNA dataset. The dataset of Walker 2012 [87] was omitted because its recurrent ^cancer^CNA regions covered 94% of the human genome, and we were concerned that this level of coverage would be overbearing.

#### ^somatic^CNV datasets

Six ^somatic^CNVs were obtained from Piotrowski *et al*. [63], Forsberg *et al*. [64], Jacobs *et al*. [65], Laurie *et al*. [66], O'Huallachain *et al*. [67], and McConnell *et al.* [68]. So as not to confound the analysis of ^somatic^CNVs with ^cancer^CNAs, all ^somatic^CNV datasets were also filtered to remove any representing individuals where a cancer-affected tissue is used to call ^somatic^CNVs. This affected two studies, Jacobs *et al.* [65] and Laurie *et al*. [66]. For Jacobs *et al.* [65], the excluded regions were from 16 patients with AML (Acute Myeloid Leukemia), CLL (Chronic Lymphocytic Leukemia), CML (Chronic Myelogenous Leukemia) or NHL (Non-Hodgkin Lymphoma) and from whom blood was used for ^somatic^CNV discovery. For Laurie *et al.* [66], the excluded regions were from 7 patients with ‘prior heamatological cancer’ and from whom blood was used for ^somatic^CNVs discovery.

#### ^iPS^CNV datasets

^iPS^CNVs were obtained from Hussein *et al.* [100] and Laurent *et al.* [98]. All datasets were culled of CNVs that were also discovered in the corresponding parental cells used to produce the iPS cells. The datasets were pooled into low passage (4 and 5), medium passage (6 through 11), and high passage (12 through 36) categories, with passage numbers chosen to ensure each category was sufficiently large for our analysis.

#### microRNAs

Since the human microRNA genomic positions were obtained with respect to genome build hg19 from ftp://mirbase.org/pub/mirbase/CURRENT/genomes/hsa.gff3, they were converted to hg18 using UCSC's liftover feature (http://genome.ucsc.edu/cgi-bin/hgLiftOver). For all analyses, we used the genomic positions of the microRNA precursor sequences, which defined regions that are larger in bp than the genomic regions producing the processed microRNAs.

### Determining depletion from or enrichment of UCEs in genomic regions of interest

Tests for depletion of UCEs from, or enrichment of UCEs in, genomic regions such as CNVs, were conducted as described in Results and our previous publications [2], [25]. We compared the observed amount of overlap in base pairs between a set of CNVs and a set of UCEs to the expected overlap, as determined by a randomly placed set of elements matched to UCEs in terms of element number and length. In particular, the elements of the matched set were placed randomly on the genome 1,000 times, and the overlap between the random elements and CNVs was calculated each time, thus producing a distribution of the randomly generated expected overlaps. To provide a measurement of the difference between the distribution of expected overlaps and the observed overlap, we reported the proportion of expected overlaps that were equal to, or more extreme than, the observed overlap. The distribution of expected overlaps was assessed for normality using the Kolomogorov-Smirnov (KS) test, and the associated KS P-value is included in all supplementary tables. Whenever the expected overlaps exhibited a normal distribution, they were compared to the observed overlap using a Z-test, wherein a significant result, together with a ratio of observed overlap to mean expected overlap (obs/exp) falling below 1.0 indicated significant depletion; a significant Z-test result and an obs/exp ratio above 1.0 indicated significant enrichment. In cases where normality was not observed, we noted this in the text and reported only the obs/exp ratio and the proportion of expected overlaps that were equal to, or more extreme than, the observed overlap. In analyses in which UCEs were segregated into exonic, intronic, and intergenic categories, random elements were drawn solely from the exonic, intronic, or intergenic portions of the genome.

### Analysis of the number of times each UCE is overlapped by the individual ^cancer^CNA datasets

We determined the total number of ^cancer^CNA datasets overlapping each of the 896 HMR-HDM-HC UCEs and report this in Table S5. For exonic and intronic UCEs, we reported the gene that contains the element. In the case of a UCE that overlapped multiple genes, both genes were recorded. The list of transcripts was obtained from the UCSC Known Genes track.

### Gene ontogeny

The tool GREAT (http://bejerano.stanford.edu/great/public/html/) was used with background set to the whole genome.

### Partial correlations

Data for genomic features of interest were obtained from the following sources: UCSC genes – UCSC known genes track build hg18; Enhancer regions – ENCODE combined genome segmentation from the ENCODE UCSC hub [95] ‘E’ (enhancer) class genomic regions for six ENCODE cell/tissue types; microRNAs – miRBase [118]; GC content – UCSC genome browser; replication timing – [96].

Analyses were performed over 10 kb, 50 kb, and 100 kb windows. Results were similar for all bin sizes, with no changes in significance for ^classical^CNVs or ^cancer^CNAs. Only the results for 50 kb bins are shown in Figure 2. Positional data were converted to a density measurement by summing the number of bases in a window covered by the feature of interest (e.g. UCE, CNV), divided by the number of sequenced bases in the hg18 human genome within the same window. Partial correlations were performed using Matlab partialcorr function.

### Genome coordinates

All coordinates listed in this study are with reference to human genome build hg18. All start coordinates are 1-based.

### Scripts

All scripts for this study are written in Python and are available at https://github.com/rmccole/Abnormal_dosage_UCEs.

## Supporting Information

Figure S1 Intersections of the CoDHo, DMR, and HMR datasets of UCEs. We defined two new datasets of UCEs without reference to the human genome, and compared them to a dataset of UCEs identified using human, mouse, and rat [1]. These datasets, CoDHo and DMR, show considerable overlap with each other and the HMR dataset. Details on the build used to identify UCEs are given in the Methods. All intersections are given in bp.
(PDF)

Table S1 CNV datasets. (A) Information on datasets. Subsequent tabs: The coordinates for each set of regions listed in (A) are contained in a tab, with the dataset name corresponding to the tab title.
(XLS)

Table S2 Depletion of UCEs from ^classical^CNVs is maintained in UCE datasets defined using different species. (A) Depletion analysis of UCEs representing the union of Human-Mouse-Rat (HMR), Human-Dog-Mouse (HDM), and Human–Chicken (HC) UCEs, as in Derti *et al*. [2], from ^classical^CNV datasets. (B) Depletion analysis of UCEs defined using the dog, mouse, and rat reference genomes from all ^classical^CNV datasets. (C) Depletion analysis of UCEs defined using the cow, dog, and horse reference genomes from all ^classical^CNV datasets. (D) Depletion analysis of UCEs defined using the human, mouse, and rat reference genomes from all ^classical^CNV datasets. (E) UCE coordinates: Coordinates in hg18 for UCE datasets.
(XLS)

Table S3 Investigation of the robustness of depletion and enrichment analyses to the genome coverage and median size of CNV datasets. A: Establishment of a lower limit for genome coverage for depletion and enrichment analyses. We were concerned that the small genome coverage of some CNV datasets would make the datasets inappropriate for our analyses, even though we had observed significant depletion of UCEs from datasets with as little as 26 Mb of genome coverage. To further explore the impact of genome coverage, we ‘shrank’ ^classical^CNV datasets by iteratively removing bases from each end of every CNV region to produce datasets with increasingly smaller CNVs and genome coverage and then assessed the modified dataset for depletion of UCEs. These tables show the effect of decreasing median CNV size and overall genome coverage (bp) of the Jakobsson 2008 [61] and Campbell 2011 [60] ^classical^CNV datasets, both of which show depletion for UCEs. Significance of depletion (P = 0.034, obs/exp  = 0.369) was retained for the Jakobsson 2008 dataset even when genome coverage was reduced to 30 Mb. However, under 20Mb, the expected overlaps were no longer normally distributed. With the Campbell 2011 dataset, depletion was maintained with all levels of genome coverage, the lowest tested being as little as 10 Mb (P = 0.042, obs/exp  = 0.000). Similarly to the Jakobsson 2008 dataset, the expected overlaps for the Campbell 2011 dataset were not consistently normally distributed when genome coverage was 20 Mb or less. Taking all these observations into account, we chose 20 Mb as the lower limit of genome coverage for our analyses. We also pooled CNV datasets together to achieve larger datasets, in which we would have more confidence. B: Analysis of enlarged^ classical^CNV datasets for UCE depletion. Reproduced from Results. Importantly, large genome coverage and CNA size are unlikely to explain enrichment or loss of depletion of UCEs in ^cancer^CNA datasets, and three findings support this statement. First, the broad range of genome coverage for ^cancer^CNA datasets showing enrichment or loss of depletion (from 90.15% for Walker 2012 ^cancer^CNAs to 3.86% for TCGARN 2012 colon ^cancer^CNAs) overlaps that for datasets that are depleted of UCEs (from 51.37% for pooled ^classical^CNVs to 0.83% for Campbell 2011 ^classical^CNVs), arguing that genome coverage alone cannot easily account for our observations of enrichment or depletion (Tables 2 and 3, S1, S2, and S4). Second, depletion is maintained when the boundaries of each CNV of the Jakobsson 2008 ^classical^CNV and Campbell 2011 ^classical^CNV datasets are extended on each side by 4.0 and 2.5 Mb to genome coverages of 85.16% and 74.73%, respectively (P  = 0.007, obs/exp  = 0.968 and P = 0.003, obs/exp  = 0.952, respectively), such that the genome coverages of these enlarged datasets approach or exceed the genome coverages of the two largest ^cancer^CNA datasets (90.15% for Walker 2012 ^cancer^CNAs and 63.81% for pooled ^cancer^CNAs), once again indicating that high genome coverage does not produce false signals of enrichment or loss of depletion (Tables S1 and S3 section B). We note, however, that as the genome coverage of the Walker 2012 ^cancer^CNA dataset is extremely high and exceeds the genome coverage of the enlarged ^classical^CNV datasets, we cannot rule out some contribution of genome coverage to the enrichment of this specific dataset. Third, these analyses also reveal that depletion is maintained even when the median length of enlarged CNVs (3.485 Mb and 8.379 Mb for Jakobsson 2008 ^classical^CNVs and Campbell 2011 ^classical^CNVs, respectively) exceeds the largest median CNA size for any enriched ^cancer^CNA dataset in question (3.183 Mb for TCGARN 2012 squamous ^cancer^CNAs), demonstrating that observations of UCE enrichment are unlikely to be explained simply by median CNA size (Tables S1 and S3 section B).
(XLS)

Table S4 Analyses of all (A) *^de nov^°*CNVs, (B) ^cancer^CNAs, (C) ^somatic^CNVs, and (D) ^iPS^CNVs.
(XLS)

Table S5 Analysis of the number of times each UCE is overlapped by the individual ^cancer^CNA datasets.
(XLS)
