# Supplementary material for: Abnormal Dosage of Ultraconserved Elements Is Highly Disfavored in Healthy Cells but Not Cancer Cells
Source: PLoS Genet. 2014 Oct 23;10(10):e1004646. doi: 10.1371/journal.pgen.1004646 (PMC4207606; doi:10.1371/journal.pgen.1004646)
Supplement: Figure S1 — Intersections of the CoDHo, DMR, and HMR datasets of UCEs. We defined two new datasets of UCEs without reference to the human genome, and compared them to a dataset of UCEs identified using human, mouse, and rat [1]. These datasets, CoDHo and DMR, show considerable overlap with each other and the HMR dataset. Details on the build used to identify UCEs are given in the Methods. All intersections are given in bp. (PDF) [file pgen.1004646.s001.pdf]

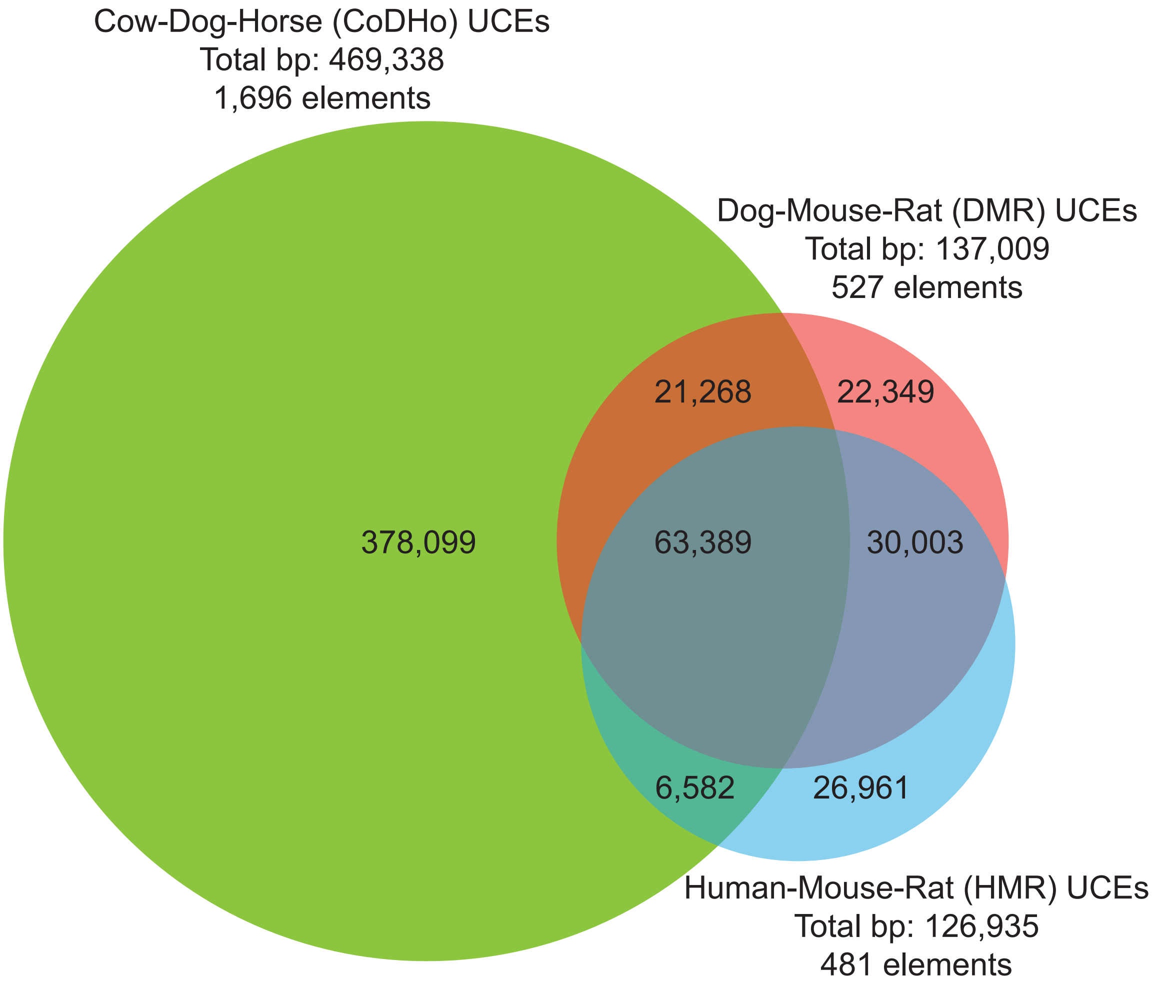

Figure S1: Intersections of the CoDHo, DMR, and HMR datasets of UCEs.
We defined two new datasets of UCEs without reference to the human genome, and compared them to a dataset of UCEs identified using human, mouse, and rat (1). These datasets, CoDHo and DMR, show considerable overlap with each other and the HMR dataset. Details on the build used to identify UCEs are given in the Methods. All intersections are given in bp.

1. Bejerano G et al. (2004) Ultraconserved elements in the human genome. Science 304:1321–1325.
